# {4,4′-Dibromo-2,2′-[2,2-dimethyl­propane-1,3-diylbis(nitrilo­methyl­idyne)]diphenolato-κ^4^ *O*,*N*,*N*′,*O*′}copper(II)

**DOI:** 10.1107/S1600536808036635

**Published:** 2008-11-13

**Authors:** Hadi Kargar, Hoong-Kun Fun, Reza Kia

**Affiliations:** a Department of Chemistry, School of Science, Payame Noor University (PNU), Ardakan, Yazd, Iran; b X-ray Crystallography Unit, School of Physics, Universiti Sains Malaysia, 11800 USM, Penang, Malaysia

## Abstract

In the title compound, [Cu(C_19_H_18_Br_2_N_2_O_2_)], the Cu^II^ ion is in a tetra­hedrally distorted planar geometry, involving two N and two O atoms from the tetra­dentate Schiff base ligand. Inter­molecular C—H⋯O hydrogen bonds form an eight-membered *R*_2_^2^(8) motif. The dihedral angle betwen two benzene rings is 36.34 (9)°. There are inter­molecular Cu⋯Br [3.4566 (5) Å] and Cu⋯·N [3.569 (3) Å] contacts, which are significantly shorter than the sum of van der Waals radii of the relevant atoms. These inter­actions, along with the inter­molecular C—H⋯π and π–π [centroid–centroid distances of 3.709 (1) and 3.968 (2) Å] inter­actions, link neighbouring mol­ecules into a one-dimensional infinite chain along the *c* axis.

## Related literature

For bond-length data, see: Allen *et al.* (1987 ▶). For hydrogen-bond motifs, see: Bernstein *et al.* (1995 ▶). For values of van der Waals radii, see: Bondi (1964 ▶). For related structures, see: Arıcı *et al.* (2001 ▶); Elmali *et al.* (2000 ▶); Hodgson (1975 ▶); Granovski *et al.* (1993 ▶). For the application of transition-metal complexes with Schiff base ligands, see: Blower (1998 ▶); Shahrokhian *et al.* (2000 ▶).
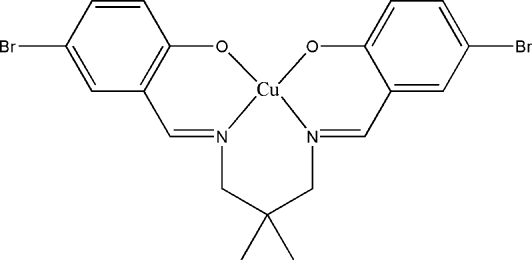


## Experimental

### 

#### Crystal data

[Cu(C_19_H_18_Br_2_N_2_O_2_)]
*M* *_r_* = 529.71
Triclinic, 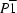

*a* = 9.1416 (3) Å
*b* = 9.6398 (3) Å
*c* = 11.5382 (3) Å
α = 75.210 (2)°
β = 78.913 (2)°
γ = 73.435 (2)°
*V* = 934.42 (5) Å^3^
*Z* = 2
Mo *K*α radiation
μ = 5.46 mm^−1^
*T* = 100.0 (1) K
0.41 × 0.21 × 0.15 mm

#### Data collection

Bruker SMART APEXII CCD area-detector diffractometer
Absorption correction: multi-scan (**SADABS**; Bruker, 2001 ▶) *T*_min_ = 0.195, *T*_max_ = 0.443
29164 measured reflections
5410 independent reflections
4345 reflections with *I* > 2σ(*I*)
*R*_int_ = 0.037

#### Refinement

*R*[*F* ^2^ > 2σ(*F* ^2^)] = 0.033
*wR*(*F* ^2^) = 0.093
*S* = 1.07
5410 reflections
235 parameters
H-atom parameters constrained
Δρ_max_ = 1.27 e Å^−3^
Δρ_min_ = −0.61 e Å^−3^

### 

Data collection: *APEX2* (Bruker, 2007 ▶); cell refinement: *SAINT* (Bruker, 2007 ▶); data reduction: *SAINT*; program(s) used to solve structure: *SHELXTL* (Sheldrick, 2008 ▶); program(s) used to refine structure: *SHELXTL*; molecular graphics: *SHELXTL*; software used to prepare material for publication: *SHELXTL* and *PLATON* (Spek, 2003 ▶).

## Supplementary Material

Crystal structure: contains datablocks global, I. DOI: 10.1107/S1600536808036635/hy2161sup1.cif

Structure factors: contains datablocks I. DOI: 10.1107/S1600536808036635/hy2161Isup2.hkl

Additional supplementary materials:  crystallographic information; 3D view; checkCIF report

## Figures and Tables

**Table 1:** Selected bond lengths (Å)

Cu1—O2	1.9027 (19)
Cu1—O1	1.9146 (18)
Cu1—N1	1.948 (2)
Cu1—N2	1.955 (2)

**Table 2:** Hydrogen-bond geometry (Å, °)

*D*—H⋯*A*	*D*—H	H⋯*A*	*D*⋯*A*	*D*—H⋯*A*
C16—H16*A*⋯O2^i^	0.93	2.44	3.342 (3)	163
C10—H10*B*⋯*Cg*1^ii^	0.97	2.50	3.324 (3)	142

Symmetry codes: (i) 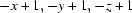; (ii) 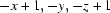. *Cg*1 is the centroid of the Cu1, N2, O2, C11, C12, C17 ring.

**Fig. 1.**
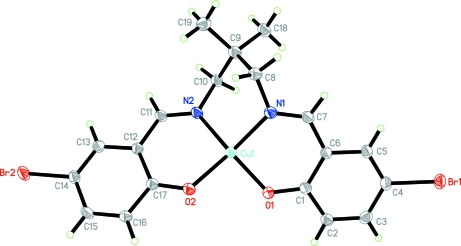
The molecular structure of the title compound, showing 50% probability displacement ellipsoids.

**Fig. 2.**
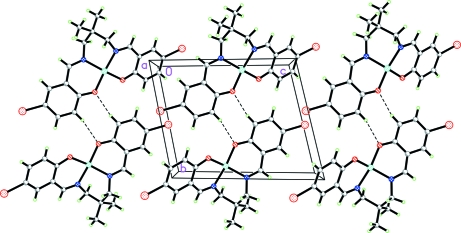
The crystal packing of the title compound, viewed down the a axis, showing the hydrogen-bond motif R22(8).

**Fig. 3.**
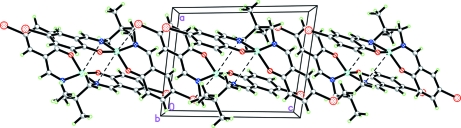
The crystal packing of the title compound, viewed down the b axis, showing one-dimensional infinite chains along the c axis. Intermolecular Cu···Br and Cu···N interactions are shown as dashed lines.

## supplementary crystallographic information

### Comment

Schiff base complexes are some of the most important stereochemical models in transition metal coordination chemistry, with their ease of preparation and structural variations (Elmali *et al.*, 2000; Granovski *et al.*,1993). Transition metal complexes of Schiff base ligands are always of interest since they exhibit a marked tendency to oligomerize, thus leading to novel structural types, and also display a wide variety of magnetic properties (Blower, 1998; Shahrokhian *et al.*, 2000). Many of the reported structural investigations of these complexes are discussed in some details in a review (Hodgson, 1975). Tetradentate Schiff base metal complexes may form *trans* or *cis* planar or tetrahedral structures (Elmali *et al.*, 2000).

In the title compound (Fig. 1), the Cu^II^ ion shows a planar geometry distorted towards tetrahedral, which is defined by two imine N atoms and two phenolate O atoms of the tetradentate Schiff base ligand (Table 1). Intermolecular C—H···O hydrogen bonds form an eight-membered ring *R*_2_^2^(8) motif (Fig. 2) (Bernstein *et al.*, 1995). The bond lengths are within the normal ranges (Allen *et al.*, 1987) and are comparable with the related structure (Arici *et al.*, 2001). The dihedral angle between two benzene rings is 36.34 (9)°. The chelate ring composed of Cu1, N1, C8, C9, C10 and N2 atoms has a distorted boat conformation with puckering paremeters of Q = 0.807 (3) Å, Θ = 91.1 (2)° and Φ = 264.58 (17)°. The interesting feature of the crystal structure is short intermolecular Cu1···Br1^iii^ [3.4566 (5) Å] and Cu1···N2^ii^ [3.569 (3) Å] interactions [symmetry codes: (ii) 1 - *x*, -*y*, 1 - *z*; (iii) 1 - *x*, -*y*, 2 - *z*], which are significantly shorter than the sum of van der Waals radii of the relevant atoms [Cu: 2.32; Br: 1.85; N: 1.55 Å (Bondi, 1964; Spek, 2003)]. These interactions along with the intermolecular C—H···π (Table 2) and π–π interactions [centroid–centroid distances: *Cg*2···*Cg*3^iii^ = 3.709 (1) and *Cg*2···*Cg*2^iii^ = 3.968 (2) Å; *Cg*2 = centroid of the C1–C6 ring and *Cg*3 = centroid of the Cu1, N1, O1, C1, C6, C7 ring] link the neighbouring molecules into one-dimensional infinite chains along the *c* axis (Fig. 3).

### Experimental

The title compound was prepared based on the reported method (Arici *et al.*, 2001). Single crystals suitable for X-ray analysis were obtained from an ethanol solution at room temperature.

### Refinement

H atoms were positioned geometrically and refined using a riding model, with C—H = 0.93 (aromatic), 0.97 (CH_2_) and 0.96 (CH_3_) Å and *U*_iso_(H) = 1.2 (1.5 for methyl groups) *U*_eq_(C). The highest difference peak is located 0.81 Å from Br2 and the deepest hole is located 0.76 Å from Cu1.

### Crystal data

**Table:**

[Cu(C_19_H_18_Br_2_N_2_O_2_)]	*Z* = 2
*M**_r_* = 529.71	*F*_000_ = 522
Triclinic, *P*1	*D*_x_ = 1.883 Mg m^−^^3^
Hall symbol: -P 1	Mo *K*α radiation λ = 0.71073 Å
*a* = 9.1416 (3) Å	Cell parameters from 9921 reflections
*b* = 9.6398 (3) Å	θ = 2.2–33.8º
*c* = 11.5382 (3) Å	µ = 5.46 mm^−^^1^
α = 75.210 (2)º	*T* = 100.0 (1) K
β = 78.913 (2)º	Block, red
γ = 73.435 (2)º	0.41 × 0.21 × 0.15 mm
*V* = 934.42 (5) Å^3^	

### Data collection

**Table:**

Bruker SMART APEXII CCD area-detector diffractometer	5410 independent reflections
Radiation source: fine-focus sealed tube	4345 reflections with *I* > 2σ(*I*)
Monochromator: graphite	*R*_int_ = 0.037
*T* = 100.0(1) K	θ_max_ = 30.0º
φ and ω scans	θ_min_ = 1.8º
Absorption correction: multi-scan(*SADABS*; Bruker, 2001)	*h* = −12→12
*T*_min_ = 0.195, *T*_max_ = 0.443	*k* = −13→13
29164 measured reflections	*l* = −16→16

### Refinement

**Table:**

Refinement on *F*^2^	Secondary atom site location: difference Fourier map
Least-squares matrix: full	Hydrogen site location: inferred from neighbouring sites
*R*[*F*^2^ > 2σ(*F*^2^)] = 0.033	H-atom parameters constrained
*wR*(*F*^2^) = 0.093	*w* = 1/[σ^2^(*F*_o_^2^) + (0.0524*P*)^2^ + 0.5947*P*] where *P* = (*F*_o_^2^ + 2*F*_c_^2^)/3
*S* = 1.07	(Δ/σ)_max_ = 0.002
5410 reflections	Δρ_max_ = 1.27 e Å^−^^3^
235 parameters	Δρ_min_ = −0.61 e Å^−^^3^
Primary atom site location: structure-invariant direct methods	Extinction correction: none

### Special details

**Table:**

Experimental. The low-temperature data was collected with the Oxford Cyrosystem Cobra low-temperature attachment.

### Fractional atomic coordinates and isotropic or equivalent isotropic displacement parameters (Å^2^)

**Table:**

	*x*	*y*	*z*	*U*_iso_*/*U*_eq_	
Cu1	0.60215 (4)	0.07295 (3)	0.63222 (3)	0.01866 (8)	
Br1	0.15151 (3)	−0.15996 (3)	1.22034 (2)	0.02498 (8)	
Br2	0.80441 (4)	0.43866 (3)	−0.00230 (2)	0.02942 (9)	
O1	0.4262 (2)	0.1481 (2)	0.73927 (16)	0.0210 (4)	
O2	0.5974 (2)	0.2636 (2)	0.53114 (17)	0.0226 (4)	
N1	0.6688 (3)	−0.1090 (2)	0.7503 (2)	0.0195 (4)	
N2	0.6996 (3)	−0.0301 (2)	0.5008 (2)	0.0193 (4)	
C1	0.3696 (3)	0.0739 (3)	0.8409 (2)	0.0187 (5)	
C2	0.2233 (3)	0.1404 (3)	0.8980 (2)	0.0216 (5)	
H2A	0.1701	0.2336	0.8604	0.026*	
C3	0.1577 (3)	0.0712 (3)	1.0073 (2)	0.0222 (5)	
H3A	0.0617	0.1176	1.0423	0.027*	
C4	0.2363 (3)	−0.0692 (3)	1.0654 (2)	0.0209 (5)	
C5	0.3765 (3)	−0.1391 (3)	1.0130 (2)	0.0202 (5)	
H5A	0.4274	−0.2325	1.0520	0.024*	
C6	0.4441 (3)	−0.0708 (3)	0.9004 (2)	0.0182 (5)	
C7	0.5935 (3)	−0.1509 (3)	0.8535 (2)	0.0195 (5)	
H7A	0.6390	−0.2402	0.9019	0.023*	
C8	0.8231 (3)	−0.1986 (3)	0.7167 (3)	0.0220 (5)	
H8A	0.8583	−0.2707	0.7877	0.026*	
H8B	0.8935	−0.1347	0.6889	0.026*	
C9	0.8276 (3)	−0.2804 (3)	0.6167 (2)	0.0215 (5)	
C10	0.7088 (3)	−0.1897 (3)	0.5308 (2)	0.0208 (5)	
H10A	0.7350	−0.2262	0.4566	0.025*	
H10B	0.6084	−0.2049	0.5677	0.025*	
C11	0.7379 (3)	0.0315 (3)	0.3905 (2)	0.0191 (5)	
H11A	0.7818	−0.0304	0.3364	0.023*	
C12	0.7183 (3)	0.1880 (3)	0.3441 (2)	0.0184 (5)	
C13	0.7662 (3)	0.2337 (3)	0.2195 (2)	0.0200 (5)	
H13A	0.8138	0.1630	0.1734	0.024*	
C14	0.7432 (3)	0.3807 (3)	0.1663 (2)	0.0206 (5)	
C15	0.6701 (3)	0.4888 (3)	0.2346 (2)	0.0221 (5)	
H15A	0.6534	0.5887	0.1975	0.026*	
C16	0.6231 (3)	0.4473 (3)	0.3559 (2)	0.0221 (5)	
H16A	0.5755	0.5202	0.3999	0.027*	
C17	0.6454 (3)	0.2960 (3)	0.4160 (2)	0.0193 (5)	
C18	0.7856 (3)	−0.4283 (3)	0.6727 (3)	0.0255 (6)	
H18A	0.7886	−0.4782	0.6098	0.038*	
H18B	0.6840	−0.4106	0.7165	0.038*	
H18C	0.8580	−0.4889	0.7268	0.038*	
C19	0.9898 (3)	−0.3061 (3)	0.5469 (3)	0.0267 (6)	
H19A	0.9944	−0.3565	0.4841	0.040*	
H19B	1.0627	−0.3653	0.6011	0.040*	
H19C	1.0142	−0.2124	0.5118	0.040*	

### Atomic displacement parameters (Å^2^)

**Table:**

	*U*^11^	*U*^22^	*U*^33^	*U*^12^	*U*^13^	*U*^23^
Cu1	0.02588 (17)	0.01331 (15)	0.01503 (15)	−0.00383 (12)	−0.00011 (12)	−0.00300 (12)
Br1	0.03447 (15)	0.02289 (14)	0.01814 (13)	−0.01260 (11)	0.00257 (10)	−0.00364 (10)
Br2	0.04858 (18)	0.01898 (14)	0.01683 (13)	−0.00907 (12)	0.00283 (11)	−0.00148 (10)
O1	0.0275 (9)	0.0161 (9)	0.0166 (9)	−0.0040 (7)	0.0010 (7)	−0.0029 (7)
O2	0.0316 (10)	0.0154 (9)	0.0182 (9)	−0.0037 (8)	0.0001 (7)	−0.0036 (7)
N1	0.0240 (11)	0.0159 (10)	0.0180 (10)	−0.0030 (8)	−0.0029 (8)	−0.0047 (9)
N2	0.0282 (11)	0.0131 (10)	0.0166 (10)	−0.0057 (8)	−0.0023 (8)	−0.0032 (8)
C1	0.0268 (13)	0.0156 (11)	0.0162 (11)	−0.0069 (10)	−0.0036 (9)	−0.0055 (9)
C2	0.0268 (13)	0.0164 (12)	0.0211 (13)	−0.0055 (10)	−0.0031 (10)	−0.0031 (10)
C3	0.0245 (13)	0.0219 (13)	0.0217 (13)	−0.0070 (10)	−0.0001 (10)	−0.0080 (11)
C4	0.0287 (13)	0.0198 (12)	0.0161 (12)	−0.0103 (10)	−0.0011 (10)	−0.0037 (10)
C5	0.0286 (13)	0.0170 (12)	0.0160 (12)	−0.0079 (10)	−0.0029 (10)	−0.0027 (10)
C6	0.0251 (12)	0.0154 (11)	0.0155 (11)	−0.0062 (9)	−0.0023 (9)	−0.0045 (10)
C7	0.0258 (13)	0.0154 (11)	0.0171 (12)	−0.0039 (10)	−0.0046 (10)	−0.0028 (10)
C8	0.0236 (12)	0.0184 (12)	0.0230 (13)	−0.0033 (10)	−0.0027 (10)	−0.0048 (11)
C9	0.0262 (13)	0.0162 (12)	0.0213 (13)	−0.0040 (10)	−0.0029 (10)	−0.0042 (10)
C10	0.0302 (13)	0.0137 (11)	0.0185 (12)	−0.0066 (10)	−0.0011 (10)	−0.0036 (10)
C11	0.0256 (12)	0.0147 (11)	0.0170 (12)	−0.0049 (9)	−0.0026 (9)	−0.0037 (9)
C12	0.0227 (12)	0.0152 (11)	0.0175 (12)	−0.0057 (9)	−0.0010 (9)	−0.0040 (10)
C13	0.0246 (12)	0.0187 (12)	0.0173 (12)	−0.0057 (10)	−0.0012 (10)	−0.0056 (10)
C14	0.0272 (13)	0.0188 (12)	0.0158 (12)	−0.0082 (10)	−0.0014 (10)	−0.0021 (10)
C15	0.0303 (14)	0.0137 (11)	0.0212 (13)	−0.0059 (10)	−0.0039 (10)	−0.0011 (10)
C16	0.0289 (13)	0.0147 (12)	0.0217 (13)	−0.0043 (10)	0.0001 (10)	−0.0056 (10)
C17	0.0232 (12)	0.0162 (11)	0.0181 (12)	−0.0052 (9)	−0.0013 (9)	−0.0038 (10)
C18	0.0361 (15)	0.0163 (12)	0.0230 (13)	−0.0064 (11)	−0.0042 (11)	−0.0020 (11)
C19	0.0269 (14)	0.0234 (14)	0.0287 (15)	−0.0048 (11)	0.0008 (11)	−0.0083 (12)

### Geometric parameters (Å, °)

**Table:**

Cu1—O2	1.9027 (19)	C8—H8A	0.9700
Cu1—O1	1.9146 (18)	C8—H8B	0.9700
Cu1—N1	1.948 (2)	C9—C18	1.530 (4)
Cu1—N2	1.955 (2)	C9—C19	1.531 (4)
Br1—C4	1.902 (3)	C9—C10	1.535 (4)
Br2—C14	1.901 (3)	C10—H10A	0.9700
O1—C1	1.305 (3)	C10—H10B	0.9700
O2—C17	1.303 (3)	C11—C12	1.437 (4)
N1—C7	1.286 (3)	C11—H11A	0.9300
N1—C8	1.467 (3)	C12—C13	1.413 (4)
N2—C11	1.287 (3)	C12—C17	1.432 (3)
N2—C10	1.470 (3)	C13—C14	1.366 (4)
C1—C2	1.422 (4)	C13—H13A	0.9300
C1—C6	1.425 (4)	C14—C15	1.405 (4)
C2—C3	1.379 (4)	C15—C16	1.373 (4)
C2—H2A	0.9300	C15—H15A	0.9300
C3—C4	1.402 (4)	C16—C17	1.420 (4)
C3—H3A	0.9300	C16—H16A	0.9300
C4—C5	1.371 (4)	C18—H18A	0.9600
C5—C6	1.411 (4)	C18—H18B	0.9600
C5—H5A	0.9300	C18—H18C	0.9600
C6—C7	1.442 (4)	C19—H19A	0.9600
C7—H7A	0.9300	C19—H19B	0.9600
C8—C9	1.544 (4)	C19—H19C	0.9600
			
O2—Cu1—O1	92.77 (8)	C19—C9—C10	110.3 (2)
O2—Cu1—N1	160.11 (9)	C18—C9—C8	110.1 (2)
O1—Cu1—N1	93.32 (9)	C19—C9—C8	108.4 (2)
O2—Cu1—N2	93.40 (8)	C10—C9—C8	110.7 (2)
O1—Cu1—N2	151.78 (9)	N2—C10—C9	113.6 (2)
N1—Cu1—N2	90.14 (9)	N2—C10—H10A	108.8
C1—O1—Cu1	126.57 (17)	C9—C10—H10A	108.8
C17—O2—Cu1	128.01 (16)	N2—C10—H10B	108.8
C7—N1—C8	119.4 (2)	C9—C10—H10B	108.8
C7—N1—Cu1	125.97 (19)	H10A—C10—H10B	107.7
C8—N1—Cu1	114.58 (17)	N2—C11—C12	125.4 (2)
C11—N2—C10	118.7 (2)	N2—C11—H11A	117.3
C11—N2—Cu1	125.90 (18)	C12—C11—H11A	117.3
C10—N2—Cu1	114.85 (16)	C13—C12—C17	120.1 (2)
O1—C1—C2	118.6 (2)	C13—C12—C11	116.7 (2)
O1—C1—C6	124.7 (2)	C17—C12—C11	123.1 (2)
C2—C1—C6	116.7 (2)	C14—C13—C12	120.7 (2)
C3—C2—C1	122.2 (3)	C14—C13—H13A	119.7
C3—C2—H2A	118.9	C12—C13—H13A	119.7
C1—C2—H2A	118.9	C13—C14—C15	120.3 (2)
C2—C3—C4	119.7 (2)	C13—C14—Br2	119.65 (19)
C2—C3—H3A	120.1	C15—C14—Br2	120.0 (2)
C4—C3—H3A	120.1	C16—C15—C14	120.2 (2)
C5—C4—C3	120.3 (2)	C16—C15—H15A	119.9
C5—C4—Br1	119.8 (2)	C14—C15—H15A	119.9
C3—C4—Br1	119.8 (2)	C15—C16—C17	121.8 (2)
C4—C5—C6	120.7 (3)	C15—C16—H16A	119.1
C4—C5—H5A	119.7	C17—C16—H16A	119.1
C6—C5—H5A	119.7	O2—C17—C16	118.9 (2)
C5—C6—C1	120.4 (2)	O2—C17—C12	124.1 (2)
C5—C6—C7	116.8 (2)	C16—C17—C12	117.0 (2)
C1—C6—C7	122.7 (2)	C9—C18—H18A	109.5
N1—C7—C6	125.3 (2)	C9—C18—H18B	109.5
N1—C7—H7A	117.3	H18A—C18—H18B	109.5
C6—C7—H7A	117.3	C9—C18—H18C	109.5
N1—C8—C9	112.7 (2)	H18A—C18—H18C	109.5
N1—C8—H8A	109.0	H18B—C18—H18C	109.5
C9—C8—H8A	109.0	C9—C19—H19A	109.5
N1—C8—H8B	109.0	C9—C19—H19B	109.5
C9—C8—H8B	109.0	H19A—C19—H19B	109.5
H8A—C8—H8B	107.8	C9—C19—H19C	109.5
C18—C9—C19	110.4 (2)	H19A—C19—H19C	109.5
C18—C9—C10	106.9 (2)	H19B—C19—H19C	109.5
			
O2—Cu1—O1—C1	−174.0 (2)	C8—N1—C7—C6	177.2 (2)
N1—Cu1—O1—C1	−12.9 (2)	Cu1—N1—C7—C6	0.6 (4)
N2—Cu1—O1—C1	83.6 (3)	C5—C6—C7—N1	176.6 (2)
O1—Cu1—O2—C17	−151.9 (2)	C1—C6—C7—N1	−6.7 (4)
N1—Cu1—O2—C17	100.4 (3)	C7—N1—C8—C9	108.8 (3)
N2—Cu1—O2—C17	0.6 (2)	Cu1—N1—C8—C9	−74.2 (2)
O2—Cu1—N1—C7	114.8 (3)	N1—C8—C9—C18	−86.0 (3)
O1—Cu1—N1—C7	7.2 (2)	N1—C8—C9—C19	153.1 (2)
N2—Cu1—N1—C7	−144.8 (2)	N1—C8—C9—C10	32.0 (3)
O2—Cu1—N1—C8	−61.9 (3)	C11—N2—C10—C9	115.7 (3)
O1—Cu1—N1—C8	−169.52 (17)	Cu1—N2—C10—C9	−72.2 (2)
N2—Cu1—N1—C8	38.50 (18)	C18—C9—C10—N2	160.8 (2)
O2—Cu1—N2—C11	0.1 (2)	C19—C9—C10—N2	−79.2 (3)
O1—Cu1—N2—C11	102.4 (3)	C8—C9—C10—N2	40.8 (3)
N1—Cu1—N2—C11	−160.3 (2)	C10—N2—C11—C12	172.3 (2)
O2—Cu1—N2—C10	−171.27 (18)	Cu1—N2—C11—C12	1.2 (4)
O1—Cu1—N2—C10	−69.0 (3)	N2—C11—C12—C13	−179.2 (3)
N1—Cu1—N2—C10	28.31 (19)	N2—C11—C12—C17	−3.2 (4)
Cu1—O1—C1—C2	−169.15 (18)	C17—C12—C13—C14	−0.2 (4)
Cu1—O1—C1—C6	11.1 (4)	C11—C12—C13—C14	175.9 (2)
O1—C1—C2—C3	−178.0 (2)	C12—C13—C14—C15	−0.6 (4)
C6—C1—C2—C3	1.9 (4)	C12—C13—C14—Br2	−178.50 (19)
C1—C2—C3—C4	0.1 (4)	C13—C14—C15—C16	0.9 (4)
C2—C3—C4—C5	−1.3 (4)	Br2—C14—C15—C16	178.8 (2)
C2—C3—C4—Br1	176.5 (2)	C14—C15—C16—C17	−0.4 (4)
C3—C4—C5—C6	0.4 (4)	Cu1—O2—C17—C16	176.52 (18)
Br1—C4—C5—C6	−177.39 (19)	Cu1—O2—C17—C12	−2.5 (4)
C4—C5—C6—C1	1.7 (4)	C15—C16—C17—O2	−179.5 (2)
C4—C5—C6—C7	178.5 (2)	C15—C16—C17—C12	−0.4 (4)
O1—C1—C6—C5	177.1 (2)	C13—C12—C17—O2	179.8 (2)
C2—C1—C6—C5	−2.7 (4)	C11—C12—C17—O2	3.9 (4)
O1—C1—C6—C7	0.5 (4)	C13—C12—C17—C16	0.7 (4)
C2—C1—C6—C7	−179.3 (2)	C11—C12—C17—C16	−175.2 (2)

### Hydrogen-bond geometry (Å, °)

**Table:**

*D*—H···*A*	*D*—H	H···*A*	*D*···*A*	*D*—H···*A*
C16—H16A···O2^i^	0.93	2.44	3.342 (3)	163
C10—H10B···Cg1^ii^	0.97	2.50	3.324 (3)	142

Symmetry codes: (i) −*x*+1, −*y*+1, −*z*+1; (ii) −*x*+1, −*y*, −*z*+1.
